# Unmanned Aerial Vehicle Cooperative Data Dissemination Based on Graph Neural Networks

**DOI:** 10.3390/s24030887

**Published:** 2024-01-30

**Authors:** Na Xing, Ye Zhang, Yuehai Wang, Yang Zhou

**Affiliations:** School of Information, North China University of Technology, No. 5 Jinyuanzhuang Road, Beijing 100144, China

**Keywords:** graph neural network, cooperative data dissemination, UAV, reinforcement learning

## Abstract

Unmanned Aerial Vehicles (UAVs) have critical applications in various real-world scenarios, including mapping unknown environments, military reconnaissance, and post-disaster search and rescue. In these scenarios where communication infrastructure is missing, UAVs will form an ad hoc network and perform tasks in a distributed manner. To efficiently carry out tasks, each UAV must acquire and share global status information and data from neighbors. Meanwhile, UAVs frequently operate in extreme conditions, including storms, lightning, and mountainous areas, which significantly degrade the quality of wireless communication. Additionally, the mobility of UAVs leads to dynamic changes in network topology. Therefore, we propose a method that utilizes graph neural networks (GNN) to learn cooperative data dissemination. This method leverages the network topology relationship and enables UAVs to learn a decision policy based on local data structure, ensuring that all UAVs can recover global information. We train the policy using reinforcement learning that enhances the effectiveness of each transmission. After repeated simulations, the results validate the effectiveness and generalization of the proposed method.

## 1. Introduction

To perform complex tasks that cannot be achieved by a single UAV, UAV swarms have gained significant attention. There are numerous practical applications, including unknown environment mapping [1], military reconnaissance [2,3], and search and rescue operations in post-disaster areas [4]. In these scenarios, communication infrastructures are often unable to be installed. Thus, UAVs need to form an ad hoc network and operate in a distributed manner.

UAV data dissemination plays a crucial role in various important application scenarios, making it a key component for UAV functionality. Its significance stems from its wide range of applications and impacts. Such as safe navigation of the UAV in unknown environments [5], intelligent transportation systems [6,7,8], and for emergency communication services in post-disaster areas [9,10,11].

**Figure 1:**
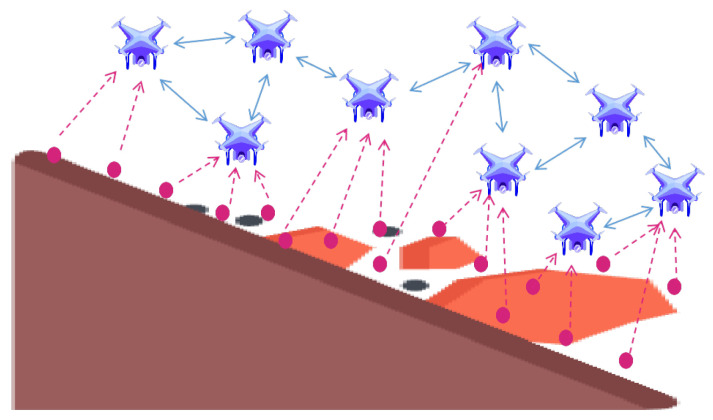
UAVs collect and disseminate data in disaster areas without communication infrastructure.

In this paper, we focus on a mapping scenario after geological disasters where UAVs play a crucial role in enhancing search and rescue efficiency. Figure 1 shows the UAVs performing data collection and data dissemination under the landslide scenario, with the pink solid circle representing data fragments (i.e., sub-maps), the pink arrow representing the current data fragments being collected by the UAV, and the blue arrow representing the communication between the UAVs.

Sub-maps are transmitted between UAVs through communication until each UAV obtains all the sub-maps. This paper aims to complete the dissemination of sub-maps in the shortest possible time; while exchanging data fragments, UAVs also exchange status information about other UAVs in the network with their neighbors.

## 2. Related Work

This section introduces the work related to this paper from recent years. Section 2.1 is the main application scenarios of data dissemination. Section 2.2 includes work that uses data encoding to improve the quality of data transmission. Section 2.3 introduces examples of using graphs to represent distributed network structures and the advantages of GNNs in processing graph-structured data.

### 2.1. Data Dissemination

Most research on data dissemination has been carried out in various vehicular ad hoc networks (VANETs) applications and requires roadside units (RSU) to feed data into the network. Yang et al. [12] propose a hybrid data dissemination model with both vehicle-to-vehicle (V2V) and vehicle-to-infrastructure (V2I) disseminations in automatic driving scenarios. The RSU injects data to the vehicles and the data disseminates via the vehicle network. For managing the driving status of the platoon, Li et al. [13] propose a method in which a lead vehicle transmits driving information to following autonomous vehicles by using multi-hop data dissemination in intelligent transportation systems.

In recent years, there has been an increasing amount of literature on data dissemination algorithms in the Internet of Things (IoT) applications regarding UAV-assisted communications [14]. To realize UAV-assisted edge computing resource scheduling for platooning vehicles in [15], Liu et al. [15] use Time Division Multiple Access (TDMA) protocol to realize the communication between UAV and the ground vehicles. Similarly, Shah et al. [16] propose a data dissemination technique using a time barrier mechanism to reduce the overhead of messages that can clutter the network. To empower the efficiency of data dissemination, Zhang et al. [17] propose a novel UAV-enabled scheduling protocol consisting of a proactive caching policy and a file-sharing strategy in V2V networks.

In this paper, there is no communication infrastructure such as RSU. Data collection and dissemination are all completed by UAVs. Therefore, the existing methods cannot be applied to the scenario proposed in this paper. We design a distributed communication algorithm for UAVs to realize data dissemination.

### 2.2. Data Coding

The performance of data dissemination can be significantly compromised by the limited bandwidth resources of UAVs. Recent studies have shown that data coding can improve bandwidth utilization. In [6], a scheduling strategy is proposed to provide efficient data dissemination with network coding and vehicular caching where infrastructures are unavailable. Ref. [18] considers the wireless network-coded video broadcast problem for users with multiple interfaces to minimize the number of transmission slots.

In this paper, we exploit packet coding to improve transmission quality.

### 2.3. Graph Representation and Graph Neural Networks

Graphs are widely utilized to illustrate UAV cooperative data dissemination. Many graph-based algorithms have been developed to facilitate UAV cooperative data dissemination. Research [19] indicates that neighbor selection based on graphs can enhance the performance of UAV cooperation. To address the broadcasting of live media streaming, Ref. [18] proposes a transmission strategy for multiple users’ mobile wireless networks. A significant step in this strategy involves finding a maximal connected subgraph within the network, giving priority to live media streaming dissemination. An investigation [20] into the code cooperative data exchange (CCDE) problem in multi-channel multi-hop wireless networks adopts the time-expanded graph approach. The CCDE addresses the recovery of desired packets in a connected network [21] and has been proven NP-hard for general topologies [22]. Inspired by the time-expanded graph, Ref. [23] resolves the CCDE problem using a conflict graph.

GNN has emerged as a powerful tool for learning representations of graph-structured data and performing various tasks on graphs. Graph Network (GN) blocks, a renowned spatial graph convolution method, define functions for relational reasoning over graph-structured representations. Graphs can express arbitrary relationships among entities, making the input to GN determine interactions between representations rather than a fixed architecture. Graphs represent entities and their relations as sets, they are permutation-invariant, rendering GNs to be unaffected by the order of elements. The per-edge and per-node functions of a GN can be reused across all edges and nodes, allowing a single GN to operate on graphs of different sizes and shapes [24].

This paper focuses on the design of a graph neural network method to realize UAV cooperative data dissemination. The method proposed in this paper includes the structural basis of the design drawing and the communication protocol, then applies the data generated by the UAV interaction to the input GNN and trains it through reinforcement learning.

The main contributions of this work are summarized as follows:

- The cooperative data dissemination problem is described in a distributed manner. We use graph structures to represent ad hoc networks and design the data structures of nodes and edges.
- This work improves wireless transmission quality through data encoding. A wireless communication protocol is designed to avoid message collision and adopts the Signal to Interference Noise Ratio (SINR) to evaluate the communication quality.
- We propose a distributed cooperative data dissemination method based on GNN. The method can adapt to the dynamic topology and enhance network efficiency and stabilization. We train the policy with a reward function that enhances the efficiency of each transmission and reduces the required number of time slots.

The remainder of this paper is organized as follows. Section 3 presents the system model and the cooperative data dissemination method is proposed in Section 4. We build the simulation and give a performance evaluation in Section 5. Finally, Section 6 concludes this work.

## 3. System Model

This section describes the system model of this work. It first performs scene modeling and symbolic expression, then introduces the input data structure in detail, and finally introduces the wireless communication model and data encoding method used in this work.

### 3.1. Scene Description

Let N={1,2,…,n} denote the UAV set. The packet set is represented as $B=\{b_{1},b_{2},\ldots b_{\left|B\right|}\}$ when there are totally |B| packets. $\kappa^{i}$ is a vector that represents serial number data packets acquired by the current UAV i∈N. It is a one-dimensional vector of length |B| with element $k^{i,m}\in \left\{0,1\right\}$, which indicates whether packet $b_{m}\in B$ is obtained by UAV *i* [25]. Let (i,j) represent a transmission link from UAV i∈N to UAV j∈N.

**Figure 2:**
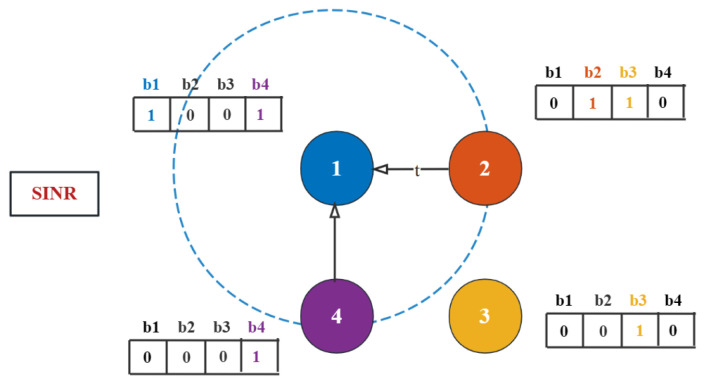
An example of UAV cooperative data dissemination consists of 4 UAVs and disseminating 4 packets.

For a better understanding, we illustrate an example as shown in Figure 2. For simplicity, we choose four UAVs holding different packets. The packet vectors of UAVs at time slot *t* are assumed as $\kappa_{t}^{1}=\left[1,0,0,1\right]$, $\kappa_{t}^{2}=\left[0,1,1,0\right]$, $\kappa_{t}^{3}=\left[0,0,1,0\right]$, and $\kappa_{t}^{4}=\left[0,0,0,1\right]$. As can be seen from this figure, UAV 1 has two neighbors, UAV 2 and UAV 4. The transmission from UAV 2 to UAV 1 can make UAV 1 obtain more packets than from UAV 4 to UAV 1. It can be seen that the number of packets in a single transmission is related to the difference between the UAVs’ packet vectors and the network topology.

### 3.2. Local Data Structure

The local data structure is the input of the proposed method. It is a combination of several feature vectors which are related to the transmission process. At time slot *t*, the local data structure of UAV i∈N is formulated as ${\left\{M_{t}^{i,j},T_{t}^{i,j},K_{t}^{i,j},L_{t}^{i,j},P_{t}^{i,j}\right\}}_{j\in N}$. It represents UAV *i*’s knowledge about the network. Primarily, $M_{t}^{i,j}$ is the status of UAV *j* known to UAV *i*. Each UAV maintains the state of other UAVs in the network, including position and velocity. And $T_{t}^{i,j}$ records the time slot when the observation of the state of UAV *j* occurs. $K_{t}^{i,j}$ is the packet vector of UAV *j* which is observed by UAV *i* after the transmission from UAV *j* to UAV *i*. The above three vectors are the attributes of UAVs. When a transmission link is established, two vectors are used to record the changes of network topology: $L_{t}^{i,j}$ denotes the time slot when transmission link (i,j) occurs. Let $P_{t}^{i,j}$ denote the first relay UAV on the path from UAV *j* to UAV *i*.

First of all, we randomly initialize the UAV attributes as the initial status of the multi-UAV system. Taking UAV *i* as an example, the state of UAV *i* is composed of the current position ${\mathrm{pos}}_{t}^{i}$ and velocity ${\mathrm{vel}}_{t}^{i}$, which are both one-dimensional vectors and changing over time. The status of UAV *i* at time slot *t* is represented as $M_{t}^{i,i}$ and the current time slot is represented as $T_{t}^{i,i}$, then:

(1) $$M_{t}^{i,i}=\left[\begin{array}{c} {\mathrm{pos}}_{t}^{i};{\mathrm{vel}}_{t}^{i} \end{array}\right],T_{t}^{i,i}=t.$$

When transmission link (j,i) occurs at time slot *t*, UAV *i* observes and records the status of UAV *j* denoted as $M_{t}^{i,j}$, the current time slot $T_{t}^{i,j}$ and the packet vector of UAV *j* observed by UAV *i* denoted as $K_{t}^{i,j}$. Then:

(2) $$M_{t}^{i,j}=M_{t}^{j,j},T_{t}^{i,j}=T_{t}^{j,j},K_{t}^{i,j}=K_{t}^{j,j}.$$

When UAV *i* receives the desired data packets, its packet vector $\kappa_{t}^{i}$ is changed. Take the example in Figure 2, $\kappa_{t}^{1}=\left[1,0,0,1\right]$ and $\kappa_{t}^{2}=\left[0,1,1,0\right]$. When transmission link (2,1) occurs, UAV 1 receives $b_{2}$ and $b_{3}$ from UAV 2. Thus, $\kappa_{t}^{1}$ is updated from [1,0,0,1] to [1,1,1,1]. At time slot *t*, the packet vector itself observed by UAV *i* is denoted by $K_{t}^{i,i}$. The time slot at which UAV *i* sends packets to UAV *j* is denoted by $L_{t}^{i,j}$.

UAVs receive physical status information about other UAVs from their neighbors and record the trajectory of the information transmission. We define the parent reference notation $P_{t}^{i,j}$ to record the destination node on the path from UAV *j* to UAV *i*, and it is recorded in UAV *i*. For example, if UAV *l* directly sends packets and UAVs’ status to UAV *i*, then $P_{t}^{i,l}=i$. It means that the parent node of the current transmission link (l,i) is UAV *l*. When UAV *i* receives UAV *l*’s status from UAV *j*, UAV *i* records the same parent reference as UAV *j*.

(3) $$K_{t}^{i,i}=\kappa_{t}^{i},L_{t}^{i,j}=t,P_{t}^{i,l}=P_{t}^{j,l}.$$

UAV *i* picks up information about other UAVs through its neighbors. When UAV *i* receives UAV *l*’s status from UAV *j*, the local data structure of UAV *i* is consistent with UAV *j*.

(4) $$\left(M_{t}^{i,l}=M_{t}^{j,l}\right)\wedge \left(T_{t}^{i,l}=T_{t}^{j,l}\right)\wedge \left(K_{t}^{i,l}=K_{t}^{j,l}\right)\wedge \left(P_{t}^{i,l}=P_{t}^{j,l}\right),\forall l\in N\backslash j.$$

By learning the knowledge of other UAVs and boosting the experience of the current UAV *i*, a connected subgraph consisting of communication links and UAVs is generated for training.

### 3.3. Communication Model

This paper has requirements for the quality of service of wireless communications. The establishment of a communication link is conditional [26,27]. We used the most common SINR to simulate wireless communications. The SINR threshold is set to a constant γ. Clearly, at time slot *t*, UAV *i* can send to UAV $j\in R_{t}^{i}$ only if the SINR value at the receiver $r_{t}^{i,j}$ satisfies:

(5) $$r_{t}^{i,j}=\frac{\theta_{t}^{i}{\left|d_{t}^{i,j}\right|}^{-\alpha }}{\eta +\sum_{k\in N\backslash i}\theta_{t}^{k}{\left|d_{t}^{k,j}\right|}^{-\alpha }}\geq \gamma .$$

where $d_{t}^{i,j}$ is the distance between UAV *i* and UAV *j* at time slot *t*, $\theta_{t}^{i}$ is the transmission power of UAV *i*, η is the noise power, and α is the pass-loss exponent. Let $R_{t}^{i}$ denote the set of UAVs that receive a transmission from UAV i∈N at time slot *t*. We can express the probability *p* of successful packet reception at UAV *j* as:

(6) $$p\left(j\in R_{t}^{i}\right)=\left\{\begin{array}{cc} 1, & r_{t}^{i,j}\geq \gamma \\ 0, & r_{t}^{i,j}<\gamma \end{array}\right.$$

A fundamental limitation of ad hoc networks with a shared medium is that the UAV can only receive at most one transmission at a time slot. Two transmissions for the same destination will result in packet collisions and no successful decoding of the data at the receiver. Additionally, in an ad hoc network, all nodes will typically compete for the same medium and therefore be able to decode any packet transmission they come within range of, regardless of whether they are the intended recipients. We allow UAVs to eavesdrop on each other’s transmissions [28].

### 3.4. Network Coding Scheme

We will use the example presented in Figure 2 to demonstrate the advantages of the coding scheme. Due to the limitation of bandwidth resources of the UAVs, we use the network coding scheme to maximize the bandwidth efficiency.

Network coding can enhance transmission efficiency, including improving throughput and reducing delay through a single coded packet which is combined by different original packets [29,30,31,32]. Let W(i) denote the packets that the UAV *i* wants and H(i) refer to the packets that UAV *i* has. W(i) and H(i) satisfy W(i)∩H(i)=∅,W(i)∪H(i)=B,i∈N. The packets transmitted by UAVs are coded with the binary sum ⊕, and the corresponding coded packet is represented as ${⊕}_{b_{b}\in H\left(i\right)}b_{b},i\in N$ [18]. To better understand the network coding scheme, we assume $B=\left\{b_{1},b_{2},b_{3},b_{4}\right\}$ and $H\left(1\right)=\left\{b_{1},b_{3}\right\}$, $W\left(1\right)=\left\{b_{2},b_{4}\right\}$, $H\left(2\right)=\left\{b_{2},b_{4}\right\}$, and $H\left(2\right)=\left\{b_{2},b_{4}\right\}$. Given a communication link (2,1), in this case, UAV 2 will transmit $b_{2}⊕b_{4}$ to UAV 1 at time slot *t*, then $H\left(1\right)=\left\{b_{1},b_{2},b_{3},b_{4}\right\}$, $W\left(1\right)=\emptyset$. According to [33], after receiving the coded packet, the receiver can instantly decode the packets it wants.

## 4. Proposed Solution

The data structure addressed in this paper is a directed graph, and it does not require connectivity. However, traditional Graph Convolutional Networks (GCNs) have certain limitations when handling non-connected graphs. These limitations arise because the convolution operations in traditional GCNs are based on the Laplacian matrix, which necessitates a connected graph. Therefore, we use a highly adaptive spatial graph neural network algorithm, which has good local perception and scalability.

Section 4.1 introduces a spatial graph neural network and gives the architecture of the method proposed by this paper. Section 4.2 introduces the transmission–response protocol and describes the data update process in detail. Section 4.3 introduces the reinforcement learning algorithm used in this paper.

### 4.1. The Local Policy with Aggregation Graph Neural Networks

Let $S_{t}^{i}$ denote the set of the receivers decided by the policy for UAV i∈N at time slot *t*. Let π denote the local policy which can give the UAV *i*’s receiver set. The policy consumes the local data structure and outputs set of receivers for each UAV. The remainder of this section introduces the π operation process in detail.

(7) $$S_{t}^{i}=\pi \left({\left\{M_{t}^{i,j},T_{t}^{i,j},K_{t}^{i,j},L_{t}^{i,j},P_{t}^{i,j}\right\}}_{j\in N}\right).$$

#### 4.1.1. Definition of Graph

First, we use a graph $G_{t}^{i}=\left\{V_{t}^{i},E_{t}^{i},u\right\}$ to represent UAV *i*’s knowledge about the ad hoc network at time slot *t*. According to the system model, the node feature set is formed as $V_{t}^{i}={\left\{M_{t}^{i,j},T_{t}^{i,j},K_{t}^{i,j},L_{t}^{i,j}\right\}}_{j\in N}$. (i,j) is a transmission link that packets successfully transmit from UAV *i* to UAV $j\in R_{t}^{i}$. Then the edge feature can be represented as $(P_{t}^{i,j},j),j\in N\backslash i$. The set of all directed edges in the graph is $E_{t}^{i}={\left\{\left(P_{t}^{i,j},j\right)\right\}}_{j\in N\backslash i}$.

#### 4.1.2. Graph Network Block

To better utilize graph-structured data, we use GN block as the main part of the policy function for reinforcement learning [24]. The input of the GN block is a graph that expresses how UAVs are isolated and interact by edges. The GN block deals nodes and edges as two sets, which means GNs are permutation invariant and the order of nodes and edges does not influence the output of GNs. The GN block uses the graph convolution operation with learnable coefficients. These coefficients equal the graph signal and multiply the powers of the adjacency matrix [34,35]. To use the GN block, we must convert the local data structure into a graph signal that can be calculated, the graph signal is represented in vectors. We flatten the node feature $\left\{M_{t}^{i,j},T_{t}^{i,j},K_{t}^{i,j},L_{t}^{i,j}\right\}$ into one-dimensional vector $v_{n}$ and flatten the edge feature $\left(P_{t}^{i,j},j\right)$ into one-dimensional vector $e_{l}$; *n* and *l* are the indexes of nodes and directed edges. Then, the local data structure is converted into graph signal $G=\{\left\{v_{n}\right\},\left\{e_{l}\right\},u\}$, u is the current time slot *t*. Define GN(·) as a function of G, which contains three parts. $\varphi^{v}$ and $\varphi^{e}$ are the update functions using original node and edge features. $\rho^{e\to v}$ is an aggregation function applied to edge features. The application of the GN block will transform original signals into $G^{\prime }=\left\{\left\{v_{n}^{\prime }\right\},\left\{e_{l}^{\prime }\right\},u\right\}$:

$$\begin{array}{c} e_{l}^{\prime }=\varphi^{e}\left(e_{l},v_{r_{l}},v_{s_{l}},u\right),\\ {\bar{e}}_{n}^{\prime }=\rho^{e\to v}\left(E_{n}^{\prime }\right), \end{array}$$

(8) $$\begin{array}{c} v_{n}^{\prime }=\varphi^{v}\left({\bar{e}}_{n}^{\prime },v_{n},u\right), \end{array}$$

where $E_{n}^{\prime }={\left\{e_{l}^{\prime },r_{l},s_{l}\right\}}_{r_{l}=n,l=1:N^{e}}$, $N^{e}$ is the number of edges; $s_{l}$ and $r_{l}$ are the sender and receiver node of edge *l*. The aggregation function $\rho^{e\to v}$ takes the set of transformed incident edge features $E_{n}^{\prime }$ at node *n* and generates the fixed-size latent vector ${\bar{e}}_{n}^{\prime }$. The Aggregation GNN updates edge and node features with learnable non-linear functions:

(9) $$\begin{array}{c} \varphi^{e}\left(e_{l},v_{r_{l}},v_{s_{l}},u\right)=NN_{e}\left(\left[e_{l},v_{r_{l}},v_{s_{l}},u\right]\right), \end{array}$$

(10) $$\begin{array}{c} \varphi^{v}\left(e_{n}^{\prime },v_{n},u\right)=NN_{v}\left(\left[e_{n}^{\prime },v_{n},u\right]\right), \end{array}$$

where $NN_{e}$ and $NN_{v}$ are both Multi-layer Perceptrons (MLPs). Moreover, the aggregation function $\rho^{e\to v}$ needs to deal with varying numbers of unordered graph signals. Thus, we need to normalize the output as follows [36]:

(11) $$\begin{array}{c} \rho^{e\to v}\left(E_{n}^{\prime }\right)=\frac{1}{\left|E_{n}^{\prime }\right|}\sum_{e_{l}\in E_{n}^{\prime }}e_{l}^{\prime }. \end{array}$$

#### 4.1.3. The Encoder-Process–Decoder Architecture

**Figure 3:**
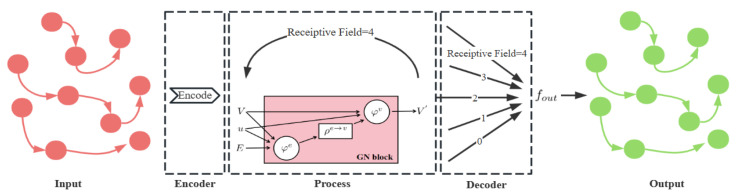
The Encoder-Process–Decoder architecture.

Inspired by [24,28], we add the encoder $f_{enc}$ and the decoder $f_{dec}$ layers on both sides of the GN layers to form the Encoder-Process–Decoder architecture which is illustrated in Figure 3. The linear output function $f_{out}$ deals a high-dimensional vector which concatenates the outputs of every GN stage [34,37], and outputs the required low-dimensional vector:

(12) $$\begin{array}{cc} \; & G^{\prime }=f_{out}\left(\left[f_{dec}\left(f_{enc}\left(G\right)\right),f_{dec}\left(GN\left(f_{enc}\left(G\right)\right)\right),\ldots \right]\right), \end{array}$$

where $f_{out}$ computes the logarithm of the Boltzmann distribution, and then generates a discrete distribution using the Gumbel-Softmax. At each time slot, each UAV samples a receiver of its transmission from this distribution. Especially, the number of GN operations determines the receptive field of GNN and how far packets can travel along edges in the network, selecting an appropriate receptive field will improve the performance of the method [38].

The receptive field refers to the specific region in the input space that a neuron or a group of neurons in a neural network is sensitive to. It is well-known that the receptive field is a critical factor for neural networks affecting performance. It determines which input signals influence the activation of the neuron or the response of the network. The receptive field can be conceptualized as a window through which the neuron or network “views” and processes information. The size and shape of the receptive field can vary depending on the architecture and parameters of the neural network.

### 4.2. Transmission–Response Protocol Design

In this section, we design a communication protocol and introduce our method in detail. The protocol is divided into two main phases: a transmission phase and a response phase. In the transmission phase, the GNN outputs recipients for each UAV and packet transfer occurs. In the response phase, the recipients of the transmission can respond. The algorithm we designed is summarized as Algorithm 1.
**Algorithm 1** Transmission–Response Protocol**Require:** $N,B,\pi ,t,{\mathrm{pos}}_{t}^{i},{\mathrm{vel}}_{t}^{i},\kappa_{t}^{i}$  1: **while** $\forall i\in N,\forall b_{m}\in B,\exists \kappa^{i,m}\;is\;0$ **do**  2:    //transmissionphasebegins  3:    $M_{t}^{i,i}:=\left[{\mathrm{pos}}_{t}^{i};{\mathrm{vel}}_{t}^{i}\right],T_{t}^{i,i}:=t,K_{t}^{i,i}:=\kappa_{t}^{i}\;\forall i\in N,t\geq 0$  4:    $S_{t}^{i}:=\pi \left(\left\{M_{t}^{i,j},T_{t}^{i,j},K_{t}^{i,j},L_{t}^{i,j},P_{t}^{i,j}\right\}\right)\;\forall i\in N$  5:    ${\left\{p(j\in R_{t}^{i})\right\}}_{i\in N}=f_{SINR}\left({\left\{S_{t}^{i},{\mathrm{pos}}_{t}^{i}\right\}}_{i\in N}\right)$  6:    $UAVs\;update\;local\;data\;structures,j\in R_{t}^{i},l\in N\backslash j$  7:    **if** $\kappa_{t}^{j,m}\;is\;0\;and\;\kappa_{t}^{i,m}\;is\;1,b_{m}\in B$ **then**  8:      $\left(M_{t}^{j,l}:=M_{t}^{i,l}\right)\wedge \left(T_{t}^{j,l}:=T_{t}^{i,l}\right)\wedge \left(K_{t}^{j,l}:=K_{t}^{i,l}\right)\wedge \left(P_{t}^{j,l}:=P_{t}^{i,l}\right)$  9:      $\left(M_{t}^{j,i}:=M_{t}^{i,i}\right)\wedge \left(T_{t}^{j,i}:=t\right)\wedge \left(K_{t}^{j,i}:=K_{t}^{i,i}\right)\wedge \left(S_{t}^{j,i}:=j\right)$ 10:    **end if** 11:    //responsephasebegins 12:    $\left(j\in R_{t}^{i}\right)\wedge \left(j\in S_{t}^{i}\right)\Rightarrow i\in \bar{S_{t}^{j}},\forall j\in N$ 13:    ${\left\{p(i\in \bar{R_{t}^{j}})\right\}}_{j\in N}=f_{SINR}\left({\left\{\bar{S_{t}^{j}},{\mathrm{pos}}_{t}^{j}\right\}}_{j\in N}\right)$ 14:    $UAVs\;update\;local\;data\;structures,i\in \bar{R_{t}^{j}},l\in N\backslash i$ 15:    **if** $\kappa_{t}^{i,m}\;is\;0\;and\;\kappa_{t}^{j,m}\;is\;1,b_{m}\in B$ **then** 16:      $\left(M_{t}^{i,l}:=M_{t}^{j,l}\right)\wedge \left(T_{t}^{i,l}:=T_{t}^{j,l}\right)\wedge \left(K_{t}^{i,l}:=K_{t}^{j,l}\right)\wedge \left(P_{t}^{i,l}:=P_{t}^{j,l}\right)$ 17:      $\left(M_{t}^{i,j}:=M_{t}^{j,j}\right)\wedge \left(T_{t}^{i,j}:=t\right)\wedge \left(K_{t}^{i,j}:=K_{t}^{j,j}\right)\wedge \left(P_{t}^{i,j}:=i\right)\wedge \left(L_{t}^{i,j}:=t\right)$ 18:    **end if** 19:    t:=t+1 20: **end while**

The communication time window *t* consists of both a transmission and a response phase. During the first half of a transmission time window, UAVs first update their local data structure with local observations. Then, the updated data structure is passed to the local communication policy, which outputs the intended recipient sets $S_{t}^{i},i\in N$. If the set consists only of the UAV itself, then no transmission occurs. UAVs then transmit to their intended recipients for their transmission.

The output set of node *i*$S_{t}^{i}$ by π may not be suitable for the wireless communication model. The function $f_{SINR}$ is defined as the communication model described in Equation (8) to evaluate whether transmission links can be successfully established. The local policy outputs the receivers of all UAVs. Then, the communication model calculates all the SINRs between UAVs and their corresponding receivers, and the output of $f_{SINR}$ is represented as $R_{t}^{i}$. The communication model is defined as:

(13) $${\left\{p(j\in R_{t}^{i})\right\}}_{i\in N}=f_{SINR}\left({\left\{S_{t}^{i},{\mathrm{pos}}_{t}^{i}\right\}}_{i\in N}\right).$$

For the transmission links that exceed the SINR threshold, the recipients will update their local data structures with new information received and record the transmission link $\left(i,j\right),j\in R_{t}^{i}$.

**Figure 4:**
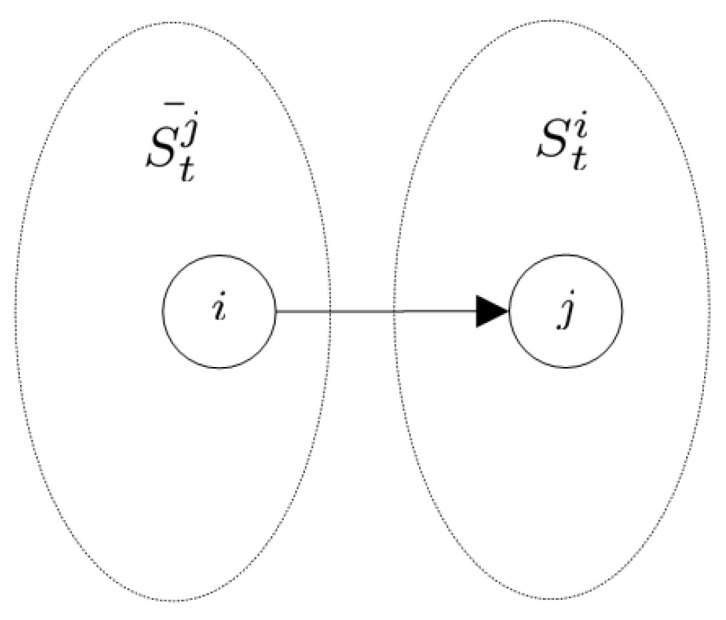
In the transmission phase, the communication link $\left(i,j\right),j\in S_{t}^{i}$ is established. When the response phase is triggered, UAV *j* responses UAV $i\in \bar{S_{t}^{j}}$.

After updating, the response phase is triggered. As shown in Figure 4, the receiver *j* of the transmission phase has a potential recipient set $\bar{S_{t}^{j}}$. After the calculation of the wireless communication model, the recipient set $\bar{R_{t}^{j}}$ of UAV *j* is determined. Then, the recipients in the transmission phase will respond to the corresponding UAVs. Again, the updating procedure of the data structure occurs.

### 4.3. Reinforcement Learning

This paper uses the Proximal Policy Optimization (PPO) method in reinforcement learning to train GNN [28,39,40], in which the policy function and the value function are the structures introduced in Section 4.1.3. $f_{enc}$, $f_{dec}$, and GN are all three-layer MLPs with 64 hidden units, and the Rectified Linear Unit (ReLU) activation is used after the first two layers. The only difference between the policy function and the value function is the output part.

In the policy function, the output function $f_{out}$ uses the Boltzmann distribution to convert high-dimensional space vectors into low-dimensional output, then uses Gumbel-Softmax to output the action probability distribution. In the value function, the output, which is a scalar, is used to evaluate the overall value.

The state space of reinforcement learning is the local data structure known by each UAV. The action space is the UAV set because all UAVs are likely to be selected as receivers in the current time slot.

#### Reward Function Design

We look forward to completing the mapping mission within a limited time frame. To minimize the time, the proportion of the number of packets obtained in a single transmission slot is used as the reward function. The more packets transmitted, the larger the step reward. To maximize cumulative rewards and reduce the total number of slots, a certain penalty is given if a packet is not successfully transmitted in a single transmission slot. The maximum reward is given when all packets are received by each UAV to encourage top-up. Hence, the reward function is designed as follows:

(14) $$Reward=\left\{\begin{array}{cc} x/X & \;\mathrm{if}\;x>0\;and\;X_{0}+\sum_{1}^{t}x<X\\ -1/X & \;\mathrm{if}\;x=0\;and\;X_{0}+\sum_{1}^{t}x<X\\ 1 & \;\mathrm{if}\;x=0\;and\;X_{0}+\sum_{1}^{t}x=X\;and\;t<\lambda \end{array}\right.$$

where x is the number of packets received by UAVs, X is the number of total packets, and $X_{0}$ is the number of packets that all UAVs have in the initial state. When x>0, the UAVs win a positive reward. λ is a constant variable that denotes the length of steps in one episode with λ=200 in this paper.

## 5. Performance Evaluation

To verify the performance of the proposed method, we design simulations in mobile scenarios. $\max_{i,j\in N,i\neq j}T_{t}^{i,j}$ represents the number of time slots required when each UAV obtains all packets. All statistical simulation results are averaged over 50 independent runs. To compare the convergence speed of reinforcement learning and the number of time slots required under different parameters, we first adjusted the settings of several parameters. Afterward, we conducted experiments under different UAV speeds, different UAV scales, and different data amounts; our method is better than the baseline algorithms.

### 5.1. Simulation Setup

Before the simulation experiment, we need to standardize some pre-variable values. We use a fixed UAV density to ensure a reasonable distance between UAVs under different sizes of UAV swarms. The UAV density is 40 UAVs per 1 km^2^ in this paper. For example, when the total number of UAVs is 20, the UAV activity area is 0.5 km^2^. The maximum sensing and communication range for UAVs is 0.25 km. At time slot *t*, we assume the velocity of UAV *i*${\mathrm{vel}}_{t}^{i}=3$ m/s and the maximum acceleration is set as 20 m/s^2^ for all experiments. We assume the communication graph is algebraic connectivity and each packet is contained by one UAV at least. In Equation (7), we assume that the pass-loss exponent α=2, the addictive white Gaussian noise η=−50 dBm, and the SINR threshold γ=1 dBm. The GNN is trained using PPO with $2\times 10^{6}$ observations. Adam optimizer is used with step size $1\times 10^{-4}$ decayed by a factor of 0.95 for every 200 steps, and a batch size of 64. Unless noted otherwise, we use a receptive field of 4 across all the below experiments.

### 5.2. Baselines

Three commonly used communication protocols are chosen to be our baselines: Random Flooding, Round Robin, and Minimum Spanning Tree (MST).

Random Flooding with a certain probability [41] is widely used in wireless communications. To balance the network load, Round Robin is also used to handle distributed network data transmission [42]. In this work, a central UAV is selected as the base station, and its neighbor exchanges information with it each time slot. The MST baseline aims to exploit the fact that MST minimizes the total edge length required to connect all UAVs in the network. It requires that the global network topology is known and the minimum spanning tree is calculated to allow interconnected UAVs to communicate with each other.

### 5.3. Simulation Results

**Figure 5:**
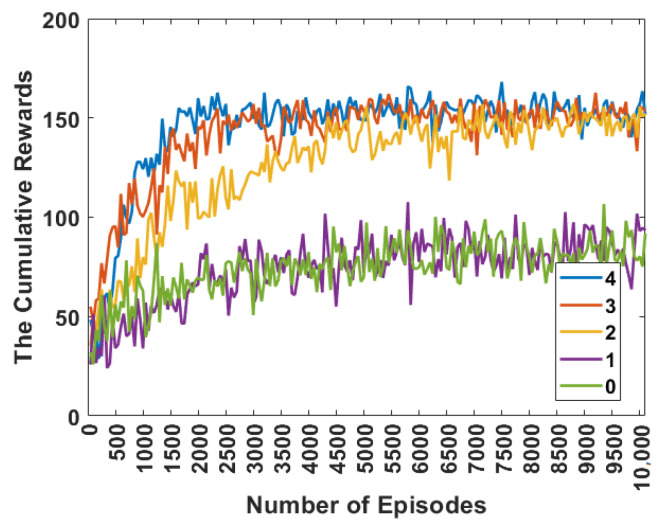
The convergence curves under different receptive fields.

To evaluate the convergence performance of our proposed cooperative data dissemination method in a mobile scenario with 20 UAVs disseminating 10 packets, Figure 5 shows the cumulative rewards with increasing training iterations under different receptive fields. The training curves are drawn to detail the statistical results of 10,200 episodes. During an episode, all UAVs run the algorithm independently and decide on receivers. This figure shows that the algorithm trains best as the receptive field increases to 4, where the cumulative reward is maximized and reinforcement learning converges fastest. From the perspective of convergence speed, the larger the receptive field is, the fewer episodes are required for convergence. This result is because as the receptive field increases, each UAV can aggregate more neighbors’ states and network topology information.

**Figure 6:**
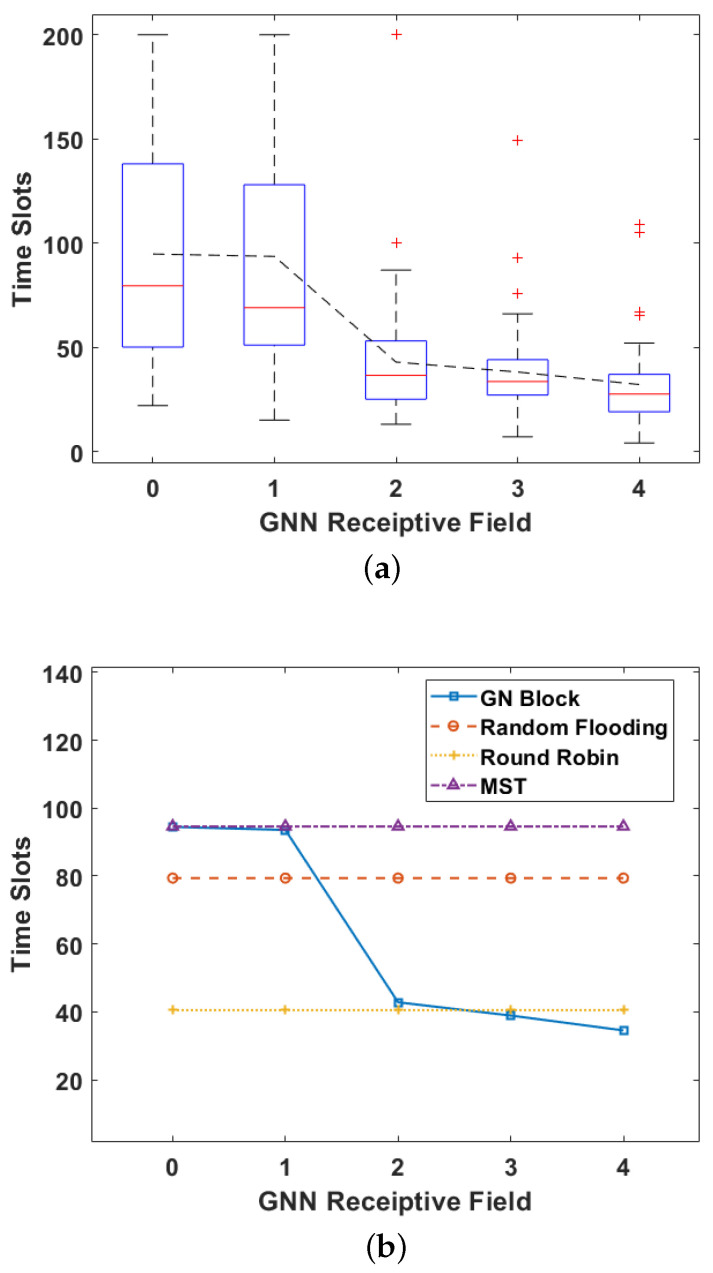
(**a**) The boxplot of the total time slots required by GN Block under different receptive fields; (**b**) The required time slots in the mobile scenario under different GNN receptive fields.

We next verify that our method requires fewer time slots than baselines. A boxplot illustrates the detail of the time slots needed under different GNN’s receptive fields in Figure 6a. The bars of the boxplot show the lowest, first quartiles, median, third quartiles, and highest values from bottom to top. This figure shows that, as the GNN receptive field grows below 4, the required number of time slots decreases. We conduct multiple experiments for each receptive field and the distribution of results is more centralized when the receptive field is 4. This is consistent with the results of the training curve. Figure 6b depicts the average time slots required with different receptive fields. Our proposed method can achieve 15% fewer time slots on average compared with the round-robin algorithm when the receptive field is 4.

**Figure 7:**
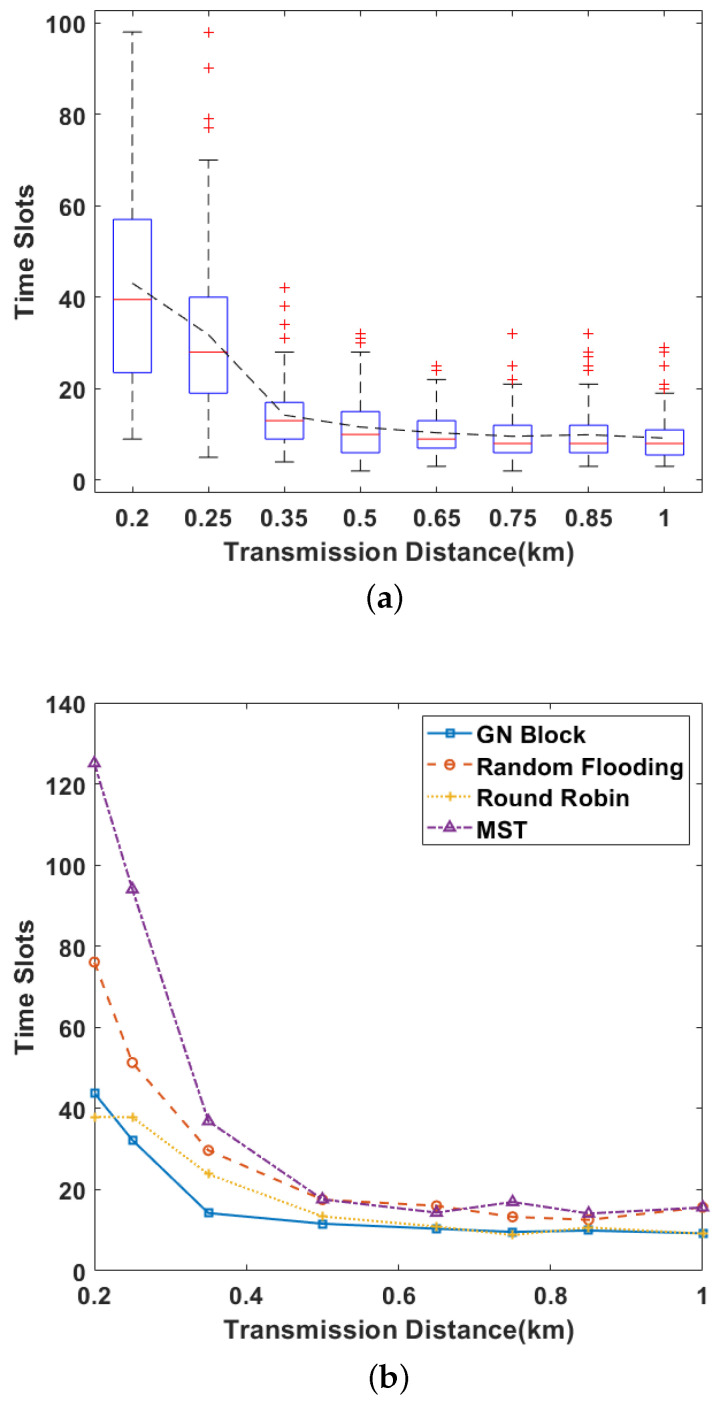
(**a**) The boxplot of the total time slots required by GN Block under different transmission distances; (**b**) The required time slots under different transmission distances.

Next, we investigate the effect of the transmission distance of UAVs. In Figure 7a, we first depict the performance of the proposed method, by evaluating the time slots required against different transmission distances. The boxplot demonstrates the distribution of the time slots required for multiple experiments with varying transmission distances. It can be seen that the required time slots decrease with the increase in transmission distances. When the transmission distance is bigger than 0.35 km, the distribution of multiple simulations’ time slots required is more concentrated. When the transmission distance is 1 km, less than 10 time slots are needed to complete data dissemination. For comparison, we also show the required time slots of three baselines. From Figure 7b, we can see that when the transmission distance is greater than 0.25 km, our method outperforms the comparison algorithms. However, UAVs often operate in harsh environments, such as storms, lightning, and mountains, which greatly affect the efficiency of wireless communication and restrict transmission distance. Therefore, the transmission distance is set as 0.25 km in this paper.

**Figure 8:**
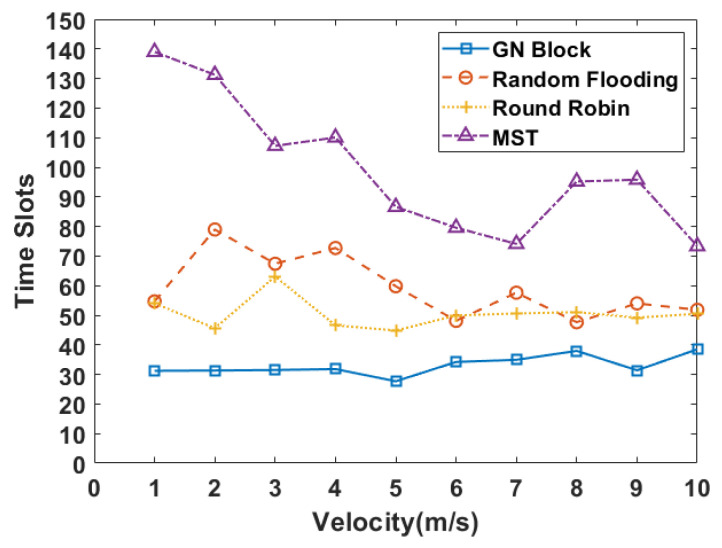
The required slots in the mobile scenario under different velocities.

Under the mobile scenario, we analyze the average time slots under different velocities. We consider a larger UAV scale. The number of UAVs is set as 40 and the number of packets is five. As can be seen from Figure 8, our method can adapt well to the mobile scenario and consumes fewer time slots than baselines with lower fluctuation, this is due to the fact that GNN is permutation invariant and the order of nodes and edges does not have an effect on the result.

**Figure 9:**
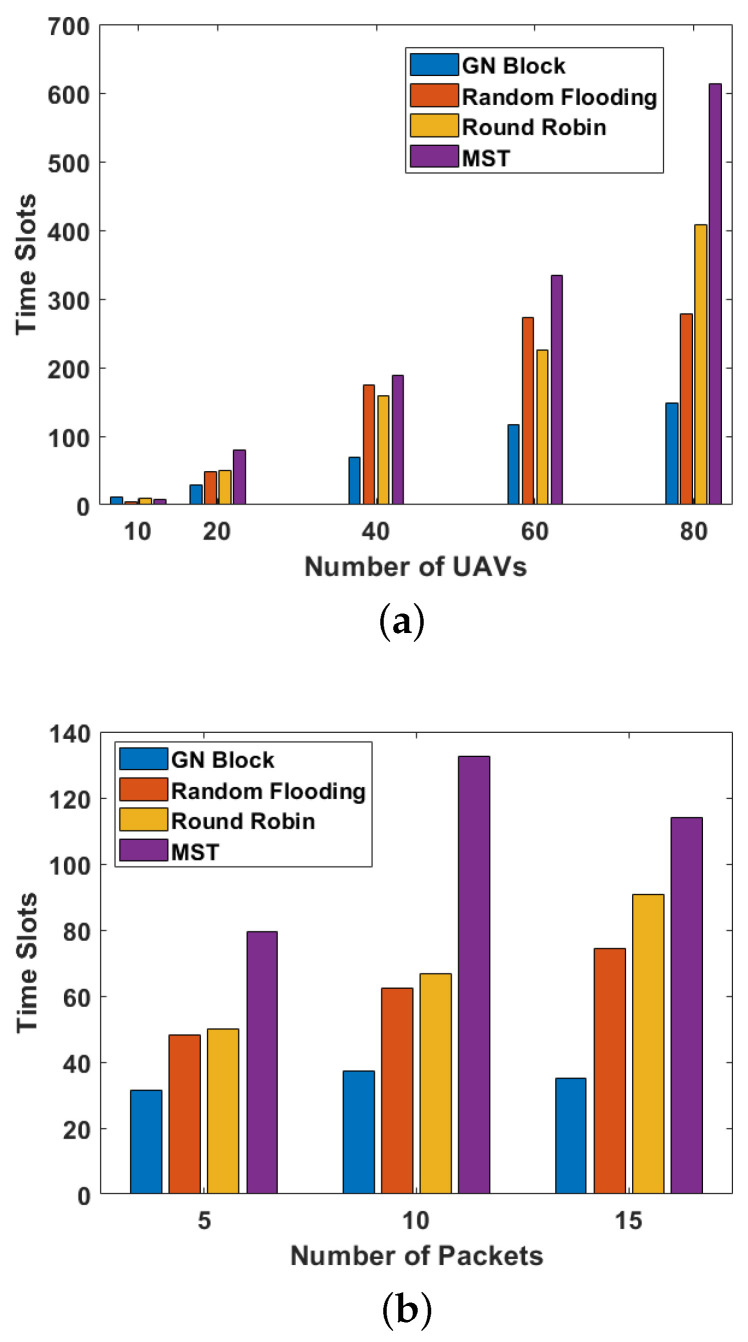
(**a**) The required slots vs. the number of UAVs; (**b**) the required slots vs. the number of packets.

Next, we evaluate the generalization of our proposed method with different numbers of UAVs and packets. Firstly, the simulation trains a model on 20 UAVs and tests it with the number of UAVs varying from 10 to 80. The number of packets is set as five. From Figure 9a, we can see that our method requires fewer time slots and performs better than all baseline algorithms when the number of UAVs is larger than 20. When the number of UAVs is more than 40, the effect of the GN Block is greatly improved compared with Robin Round. This indicates that our method will perform well when it extends to larger UAVs. Then, we evaluate the required time slots under different numbers of packets. The number of UAVs is set as 20. From Figure 9b, we can see that our method outperforms all the baseline algorithms. These figures demonstrate the effectiveness of our algorithm in mobile scenarios.

**Figure 10:**
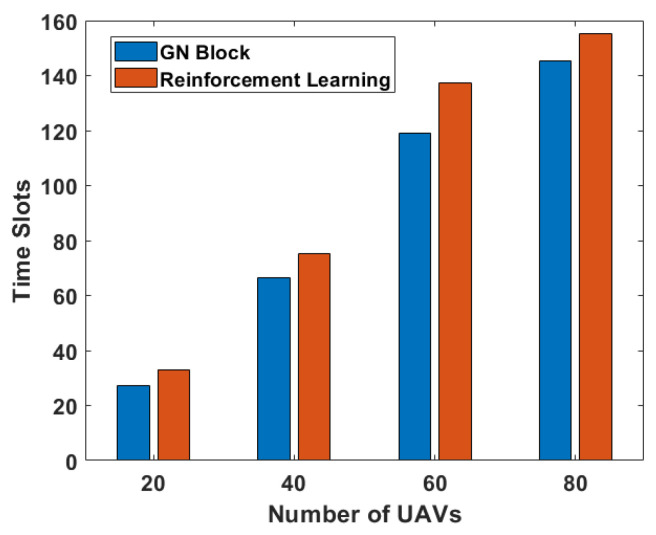
The number of time slots required by the GN block and reinforcement learning are compared, respectively, under different UAV scales.

We can observe from Figure 10 that under the condition that the total number of data packets needed to be transmitted is five, our method is more effective than the reinforcement learning method after repeated trials in different UAV scales. The experimental results demonstrate that GNN helps reduce the total data dissemination time.

## 6. Conclusions

In this paper, we propose a cooperative data dissemination method for the mapping task in searching and rescuing scenarios. Then, we propose a decision policy based on GNN. The policy determines which UAVs will communicate with each other. A wireless communication protocol is designed to constrain data forwarding. The policy is trained by reinforcement learning with a reward function designed according to the completion progress of data dissemination. Simulations show that our method outperforms all the baseline algorithms in mobile scenarios. Meanwhile, our method has great generalization. The method proposed in this paper can achieve rapid data dissemination in various distributed networks, including multi-smart vehicle space exploration, mobile user live broadcast data transmission, and scenarios such as IoV security and formation. The GNN applied in this paper can adopt certain strategies to increase the depth and further improve the experimental effect.

## Data Availability

Data are contained within the article.

## Abbreviations

The following abbreviations are used in this manuscript:

**Symbol**	**Definition**
N	the UAV set
B	the packet set
|B|	the number of packets in packet set B
$b_{m}$	the *m* th packet
$\kappa_{t}^{i}$	the packet vector of UAV *i* with element $k_{t}^{i,m}\in \left\{0,1\right\}$ at time slot *t*, $b_{m}\in B$
(i,j)	a transmission link from UAV i∈N to UAV j∈N
$d_{t}^{i,j}$	the distance between UAV i∈N and j∈N at time slot *t*
η	the noise effect
α	the pass-loss exponent
$\theta_{t}^{i}$	the transmission power of UAV i∈N
γ	the SINR threshold
$r_{t}^{i,j}$	the SINR value of the transmission link (i,j) at time slot *t*
H(i)	the packet set UAV i∈N has
W(i)	the packet set UAV i∈N wants
${\mathrm{pos}}_{t}^{i}$	the position of UAV i∈N at time slot *t*
${\mathrm{vel}}_{t}^{i}$	the velocity of UAV i∈N at time slot *t*
$M_{t}^{i,j}$	the state of UAV j∈N known by UAV i∈N at time slot *t*
$T_{t}^{i,j}$	the time slot for the observation of the state of UAV *j*
$P_{t}^{i,j}$	the first relay on the way from UAV j∈N to UAV i∈N at time slot *t*
$L_{t}^{i,j}$	the time slot when (i,j) occurs at time slot *t*
$K_{t}^{i,j}$	the packet vector of UAV j∈N observed by UAV i∈N at time slot *t*
G	graph
V	node
E	edge
π	the policy function
$v_{n}$	the *n* th node attributes
$e_{l}$	the *l* th edge attributes
u	the global attributes
$\varphi_{v}$	the update function of node attributes
$\varphi_{e}$	the update function of edge attributes
$\rho^{e\to v}$	the aggregation function which aggregate the edge features to node
$R_{t}^{i}$	the receiver set of UAV i∈N at time slot *t*
$S_{t}^{i}$	the set of the receiver set decided by the policy for UAV i∈N at time slot *t*
X_0_	the number of packets that all UAVs have in the initial state
X	the total number of packets that all UAVs should have
x	the number of packets received by UAVs in a single slot
